# A Survey on Ground Segmentation Methods for Automotive LiDAR Sensors

**DOI:** 10.3390/s23020601

**Published:** 2023-01-05

**Authors:** Tiago Gomes, Diogo Matias, André Campos, Luís Cunha, Ricardo Roriz

**Affiliations:** Centro ALGORITMI/LASI, Escola de Engenharia, Universidade do Minho, 4800-058 Guimaraes, Portugal

**Keywords:** autonomous driving, LiDAR, perception system, ground segmentation, survey

## Abstract

In the near future, autonomous vehicles with full self-driving features will populate our public roads. However, fully autonomous cars will require robust perception systems to safely navigate the environment, which includes cameras, RADAR devices, and Light Detection and Ranging (LiDAR) sensors. LiDAR is currently a key sensor for the future of autonomous driving since it can read the vehicle’s vicinity and provide a real-time 3D visualization of the surroundings through a point cloud representation. These features can assist the autonomous vehicle in several tasks, such as object identification and obstacle avoidance, accurate speed and distance measurements, road navigation, and more. However, it is crucial to detect the ground plane and road limits to safely navigate the environment, which requires extracting information from the point cloud to accurately detect common road boundaries. This article presents a survey of existing methods used to detect and extract ground points from LiDAR point clouds. It summarizes the already extensive literature and proposes a comprehensive taxonomy to help understand the current ground segmentation methods that can be used in automotive LiDAR sensors.

## 1. Introduction

In the very near future, autonomous vehicles with full self-driving features will circulate on our public roads [1]. Nonetheless, most intelligent vehicles today are still manually controlled, corresponding to the first three levels of driving automation defined by the society of automotive engineers (SAE), i.e., the six levels of automation required to reach full driving automation features. According to the SAE J3016 standard (rev. 202104) [2], in levels 0, 1, and 2, the driver must actively monitor the driving activities, while in levels 3, 4, and 5, the automated vehicle should be able to monitor and navigate the environment, autonomously. Despite current cars supporting only features up to SAE-level 2, e.g., modern advanced driver-assistance systems (ADAS) can already provide partial vehicle’s automation, car manufacturers are only now receiving approvals for SAE-level 3 [3,4], which defines explicitly that the automated driving function can take over certain driving tasks. However, a driver is still required and must be ready to take control of the car at all times when prompted to intervene by the vehicle. Achieving safe automated driving features requires robust and reliable perception systems [5] that depend on multiple sensor setups to navigate the environment, which usually include cameras, RADAR devices, and Light Detection and Ranging (LiDAR) sensors [6,7,8]. For instance, the German Federal Motor Transport Authority (KBA) has finally granted the first SAE-level 3 and UN Regulation number 157 [4] approval to Mercedes-Benz [3], whose perception system includes a wetness sensor in the wheel, LiDAR sensors, microphones, and a camera in the rear window, primarily used for detecting blue lights and other special signals from emergency vehicles. This technical approval was mainly possible due to the adoption of LiDAR to navigate the environment.

A LiDAR sensor works by firing a laser pulse to a target and capturing the reflected signal, where the distance to the target is obtained by calculating the round-trip time of the traveling light. Its output is a 3D point cloud that can be used for terrain mapping and recreating the surrounding environment. Thus, this technology has become widely popular in several airborne laser scanning (ALS) applications, such as archaeology [9,10,11], geology [12], forestry [13,14], geography and topography [14,15,16,17,18], surveying [14], laser altimetry [19], and many more [14]. Due to its wide success in such domains, LiDAR sensors recently started to be adopted by the automotive industry in the perception system of the car. Their 3D point cloud can be very useful in several autonomous driving applications [20,21,22], such as obstacles, objects, and vehicles detection [23,24,25,26]; pedestrians recognition and tracking [27,28]; ground segmentation for road detection and navigation [29]; among others [30]. Because this technology works with active illumination, LiDAR sensors allow round-the-clock observations, providing accurate measurements of the vehicle’s vicinity up to hundreds of meters. However, several challenges may affect the processing of the received point cloud such as LiDAR mutual interference [31,32,33], and adverse weather [34,35,36,37,38]. Additionally, because a high-resolution sensor can produce a considerable amount of data, e.g., the Velodyne sensor VLS-128 can output up to 9.6M points per second, it is important to handle the point cloud before being delivered to high-level applications, both in terms of packet handling [39], data compression [40,41], and point cloud denoising [36].

Regarding autonomous navigation capabilities, the vehicle must perceive the surroundings by understanding the location and shape of the driving environment. Thus, road boundaries, such as curbs, asphalt berms, walls, and other geometric features, are typical road characteristics that the perception system must automatically detect to navigate the environment safely [42]. However, detecting such features requires the perception system to perform ground and object segmentation techniques to efficiently identify or remove undesired object data [23,43,44,45]. Because this topic has been thoroughly studied in the last few years, there is a plethora of ground segmentation methods in the literature that can be used to extract road boundary information from the point cloud. Because such methods can follow different approaches, there are several trade-offs, e.g., accuracy, performance, computing requirements, etc., that must be considered before choosing the best and the most appropriate method to deploy in the perception system.

To help in understanding the existing ground segmentation methods in the literature, the main contributions of this article are as follows: (1) a literature review on state-of-the-art approaches to detect and extract ground points from LiDAR data, applied in an automotive scenario; (2) a comprehensive taxonomy proposal with five high-level categories, followed by a discussion of the technical aspects and algorithms of the approaches that can currently be found in the literature; (3) and a qualitative comparison between the different categories regarding important metrics, including real-time, resource requirements, and the algorithm’s performance in different environments and aspects. The remainder of this article is organized as follows: Section 2 presents the concepts and applications behind LiDAR, while Section 3 shows current ground segmentation methods and proposed taxonomy. Section 4 discusses and gives a qualitative comparison between the state-of-the-art contributions. Finally, Section 5 concludes this article.

## 2. Automotive LiDAR Sensors

**Figure 1:**
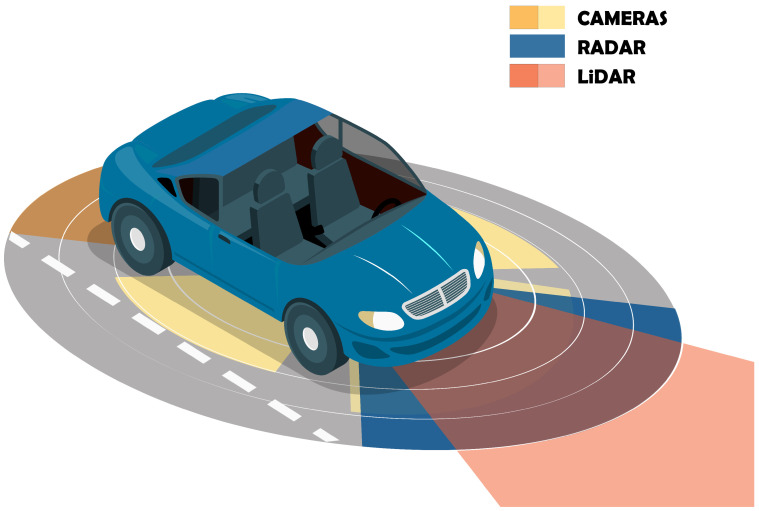
Multi-sensor perception system.

For the last 20 years, autonomous driving has been a well-established research topic. However, despite some prototypes being already available and running, the automotive industry is only now starting to provide commercial products, which will soon turn autonomous driving vehicles mainstream. Aiming at reaching SAE-level 5, vehicles are being equipped with several ADAS features, which are constantly being improved every year. This pushes research to advance in different sensing technologies and in creating more robust perception systems. Figure 1 depicts a typical multi-sensor perception system composed of cameras, RADAR, and LiDAR sensors, which can help in providing cross-traffic alerts, blind spot assist [46], Adaptive Cruise Control (ACC) [47], pedestrian detection and avoidance [48], and Automatic Emergency Braking (AEB) [49,50]. Together, all these sensors allow retrieving redundant information about the surrounding environment, ensuring that high-level autonomous driving decisions are made based on accurate representations of the vehicle’s vicinity.

Among all sensors, LiDAR is becoming the most important in autonomous applications as it can provide up to 300 m of real-time 3D information about the vehicle’s surroundings. Compared with RADAR, which shares the same working principle of measuring distances based on the round-trip time of an emitted signal, LiDAR can operate in much higher frequencies due to the light properties. Moreover, while RADAR sensors only provide angular resolutions of at most 1 degree, which is insufficient for object shape estimation [51], LiDAR sensors can achieve resolutions of tenths of degrees. Thus, their output can be used for object segmentation rather than solely object detection. Nonetheless, because LiDAR sensors use light, they still present disadvantages when compared with radio-based sensors. Since light has high absorption in water, adverse weather conditions, such as heavy rainfall, can affect the overall performance of LiDAR. On the other hand, the higher wavelengths used in RADAR can present good performance in poor weather, making this sensor capable of covering longer distances but with less emitted power.

Regarding the resolution of the output data, cameras can achieve colored and high-resolution information about the driving environment. However, they are subject to several light-related issues, e.g., they cannot corrrectly work at night or in the presence of intense light sources, which can turn the segmentation tasks relying solely on cameras quite challenging. Additionally, depth information about the environment is only possible with stereo cameras and further image processing, which affects the distance measurements.

### 2.1. LiDAR Technology

**Figure 2:**
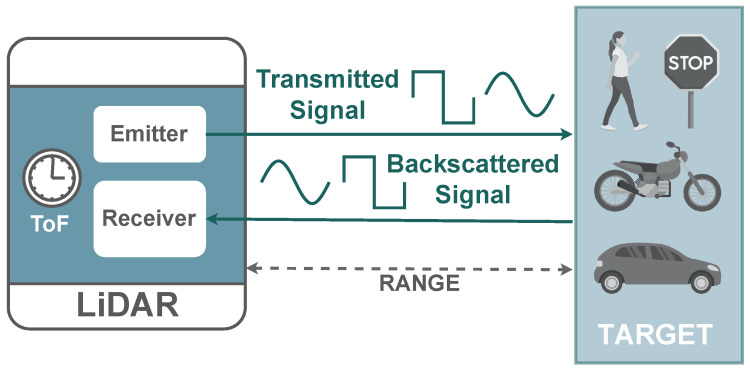
LiDAR working principle.

There is a plethora of LiDAR sensors currently available on the market [21,52]. Despite all sharing the same light-based operation properties, manufacturers already provide different measurement and imaging systems approaches, which translates into different performance metrics and overall costs. As depicted in Figure 2, a LiDAR sensor uses a light signal to measure distances, which are calculated based on the round-trip delay (τ) between a signal emitted by a laser and reflected by a target. Since the speed of light (*c*) is previously known, the distance (*R*) to the target is calculated using Equation (1).

(1) $$R=\frac{1}{2}c\tau$$

#### 2.1.1. Measurement Techniques

Depending on the sensor’s technology, the round-trip delay, also known as the Time-of-Flight (ToF), can be measured through different techniques [53,54]: pulsed, Amplitude Modulated Continuous Wave (AMCW), and Frequency Modulated Continuous Wave (FMCW). Measurement techniques based on pulsed signals are more straightforward to implement as they only require accurate timers or Time-to-Digital Converters (TDC) to directly measure and calculate the ToF. Due to their simplicity, they offer a low-cost and small-size implementation but are more subject to low Signal-Noise Ratio (SNR), which limits the accuracy of the measurements. With AMCW and FMCW, instead of sending short high-intensity pulses, the sensor emits a modulated continuous wave signal, consequently achieving better SNR values. In an AMCW system, the sensor emits a wave synchronized with a series of integrating windows. Since the reflected wave is expected to be out of phase, the distance can be calculated based on the energy ratio present on each window: short distances result in a reflected signal more present in the first windows, while for long distances, the reflected signal can be found in the last windows.

Similarly to pulsed-based sensors, AMCW can also provide a simple design. However, long-range detection requires longer wavelengths to ensure that the phase difference between signals does not exceed the signal’s period, which would produce distance ambiguity. Therefore, their application is limited mainly to mid- and short-range sensors due to eye safety regulations. On the other hand, FMCW systems modulate the emitting signal frequency with an up-chirp signal that increases its frequency over time. Then, the sensor computes the difference between the frequency of the return signal and a local oscillator, being this delta directly proportional to the distance to the target. Despite providing more robustness to external light sources and even allowing to directly retrieve the speed of a moving target due to Doppler shift, the increase in optical components makes FMCW-based sensors more expensive to build.

#### 2.1.2. Imaging Techniques

To create a 3D point cloud representation in real time, LiDAR sensors emit and collect signals across multiple directions within their supported Field of View (FoV), which can result in a point cloud holding millions of points. This is achieved by using different imaging techniques [55], such as solid-state, rotor-based, and flash-based sensors. Velodyne pioneered rotor-based LiDAR sensors. By having a mechanical system that spins the scanning part, they can create a 360º horizontal FoV, while the number of existing emitter/receptor pairs, also known as channels, define the vertical FoV. This technology was so successful that today it is the most used in LiDAR, which resulted in several manufacturers competing in this market niche. However, they still come with some limitations, such as price, bulkiness, and the reduced frame rate caused by mechanical parts.

Regarding the existing solid-state scanning solutions, some technologies, e.g., mirror MEMS [56] and Optical Phased Array (OPA) [57], are being deployed to replace the bulky rotational parts existing in rotor-based sensors. Despite achieving faster scanning frequencies and offering a better overall design for mass production (which reduces the overall product price), the sensor’s range is still a limitation due to power restrictions in the laser unit. Additionally, for applications requiring larger FoV, multiple sensors must be attached to the setup to guarantee the full coverage of the surrounding environment. Aiming at reducing the limitations created by complex bulky, or tiny steering systems, a flash-based LiDAR sensor does not require any steering system to emit laser signals across the FoV. Instead, it uses a flash of light to illuminate the entire environment while photo-detectors collect the back-scattered light. By simultaneously sharing the light source across all the FoV, capturing data results in a faster operation, making these sensors highly immune to light distortion [58]. However, despite the simplicity of the emitter, the receiver system is quite complex since it must be able to differentiate the returning light from each point. In order to guarantee a suitable sensor spatial resolution, these sensors require high-density photo-detection arrays, which makes them more expensive when compared with other solid-state solutions.

### 2.2. LiDAR Applications

LiDAR is a key sensor in a perception system since its output can be used for improving autonomous driving tasks based on sensor fusion processing [59], e.g., object detection and segmentation for object classification and collision avoidance [23,24,25], Simultaneous Localization and Mapping (SLAM) applications [60], the detection and navigation of the drivable area [61,62], and much more. The LiDAR output is a point cloud, which is an exact 3D image of the environment captured by the sensor. Humans do not easily understand its information at first sight, but algorithms can do a great job of interpreting its content.

#### 2.2.1. Object Detection and Classification

There are many methods and algorithms for detecting and classifying objects in point cloud data, but first, it is necessary to find them. After converting raw data into a point cloud structure, one of the first steps is the point clustering or segmentation, which basically consists in grouping points based on common characteristics [23]. After this step, redundant data can be filtered/removed from the point cloud, resulting in less data to be transferred and processed in the upcoming phases. In applications where the sensor keeps a stationary position, some algorithms start by classifying the point cloud into background and foreground data [24,25]. Points that share the same position across multiple frames are considered background, being discarded as they do not represent dynamic objects. For the remaining points (foreground), the distance between points is measured, and points close to each other are clustered and marked with a bounding box as they possibly represent an object. However, when the sensor moves with the car, these approaches are not effective, as the background and objects move together inside the point cloud. Therefore, automotive approaches require robust and faster algorithms since the objects in the point cloud also change at higher frequencies. First approaches applied hand-crafted features and sliding windows algorithms with Support Vector Machine (SVM) classifiers for object identification, but soon were replaced by other improved methods such as 2D representations, volumetric-based, and raw point-based data, which deploy machine learning techniques in the perception system of the vehicle [28].

#### 2.2.2. SLAM

SLAM, a well-established research topic in the field of robotics that studies solutions to build real-time localization maps based solely on perception data, has also been proposed to be applied in autonomous vehicle applications, even despite the considerable amount of 3D information the LiDAR sensor generates. Usually, odometry uses data from several sensors, e.g., IMUs and cameras, to estimate the vehicle’s position relative to a starting location. However, because LiDAR sensors can generate high data rates, some approaches follow the trend of LOAM [60], which processes the odometry task at a higher frequency, and the mapping at a much lower rate to keep the real-time requirements. Despite providing good results, they tend to suffer from problems associated with accumulated drift. To minimize this issue, some methods apply an extra re-localization step, either using offline map information to improve the position estimation, or by combining data from other sensors. Some learning-based approaches are also emerging, which can use a pipeline of Convolutional Neuronal Networks (CNNs) to manage local feature descriptors, infer the localization offset, and apply temporal smoothness due to the sequential nature of the localization task. These require, however, more computational requirements.

#### 2.2.3. Drivable Area Detection

In autonomous vehicle navigation, detecting the drivable area is one of the most critical tasks. For the car to safely move in the environment, it is necessary the detection of not only obstacles such as pedestrians or other vehicles but also several road features such as road boundaries, i.e., curbs, asphalt berms, walls and other geometric features, crosswalks, sidewalks, traffic signs, etc. [61,62]. Since LiDAR sensors can provide intensity information in the point cloud, it becomes easier to identify high-reflection road elements such as traffic signs and road marking paint. Nonetheless, to reduce data transfer, it is also necessary to distinguish background from foreground data, including ground. By classifying point cloud data into ground and non-ground points, the drivable area can be detected more efficiently since there are less points to process, which improves the navigation features of the autonomous car while keeping the real-time requirements.

## 3. Ground Segmentation Methods

**Figure 3:**
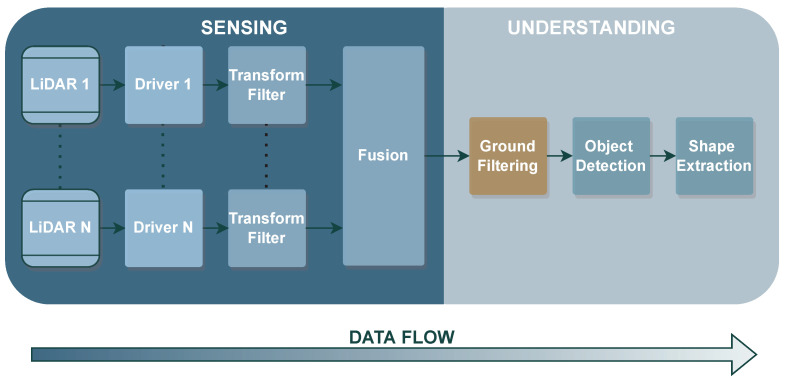
LiDAR processing stack.

Ground segmentation methods have been thoroughly studied in the literature since they play a crucial role in different autonomous driving tasks. With the evolution of LiDAR and its endless applications, several algorithms and approaches have emerged throughout the years. Figure 3 depicts the traditional data flow of a classical 3D object detection stack, which is typically simpler, faster, and requires fewer dependencies [63].

**Figure 4:**
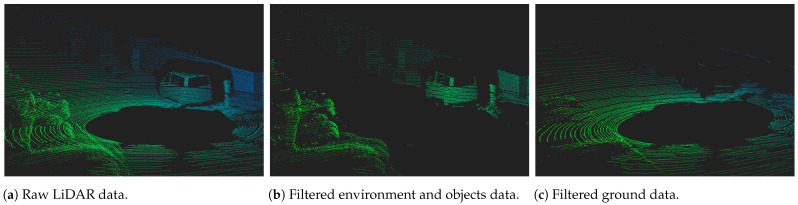
Basic principle of the ground segmentation task.

**Figure 5:**
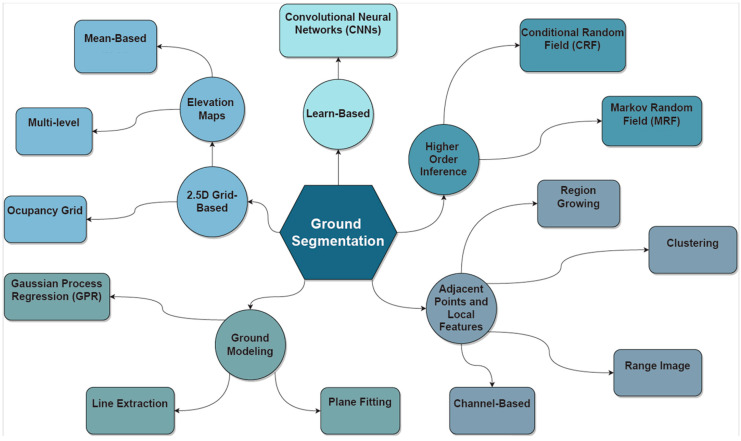
Classification and taxonomy of existing ground segmentation methods.

This stack includes the following steps in the sensing and understanding operations: (1) the software drivers translate raw data retrieved from the sensor into structured 3D point cloud data; (2) next, data is statistically transformed/filtered to remove possible noise or undesired points from the point cloud; (3) data fusion can be used to create a unique representation of the vehicle’s surroundings; (4) a ground filtering step is applied to isolate ground points from non-ground data; (5) the object detection task identifies objects in the point cloud data; and, finally (6) there is a shape extraction step to enable and perform the object classification. Regarding the ground segmentation task, its basic concept can be found in Figure 4, where the raw data collected from the LiDAR is processed to separate the points representing the vehicle’s surrounding environment from the ground points. According to the subsequent tasks, filtered data can be used for the vehicle to safely navigate the environment, or for the object detection and classification steps. In this paper, we study most of the relevant contributions that could be found in the state-of-the-art for the ground filtering step, resulting in the taxonomy illustrated in Figure 5.

Current algorithms can be classified into five different categories: (1) 2.5D Grid-based algorithms, which can be further divided in Occupancy Grids and Elevation Maps (that further include Multi-level and Mean-based algorithms); (2) Ground Modelling, which can use Gaussian Process Regression (GPR), Line Extraction, and Plane Fitting approaches; (3) Adjacent points and Local features, which include Channel-based, Range Image, Clustering, and Region Growing methods; (4) Higher Order Inference, which deploy methods based on Conditional Random Field (CRF) and Markov Random Field (MRF) approaches; (5) and Learn-Based algorithms, which mainly apply CNNs to identify and perform the ground segmentation tasks.

### 3.1. 2.5D Grid-Based

Currently, modern LiDAR sensors may hold thousands of points in their 3D point cloud representations, which makes it harder to analyze the entire point cloud in real time during the navigation tasks. To help in mitigating these problems, grid-based techniques use a tessellated 2D representation of the 3D space, where each cell contains information about the points inside. The utilization of this technique can drastically reduce the computational and memory requirements associated with the 3D point cloud representation.

**Figure 6:**
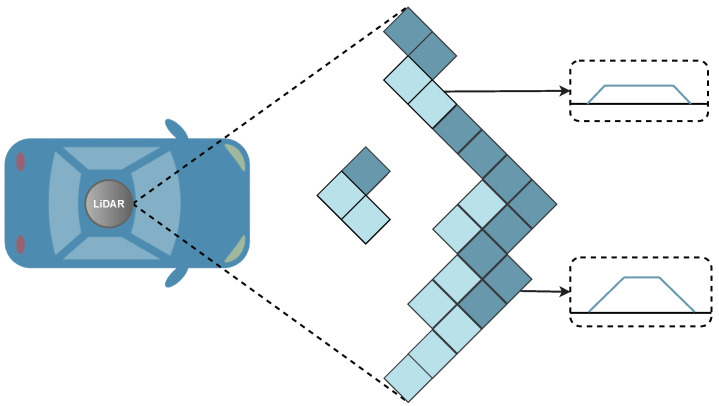
Visual representation of an Elevation Map.

***Elevation Maps:*** This technique is the most used in 2.5D grid representations. As depicted by Figure 6 (top view representation of the surrounding environment), each cell contains relevant information about all points inside. Elevation maps can provide advantages in terms of noise reduction when compared with other methods. However, they still face problems in terms of vertical space representation as they fail to model the empty space between points, i.e., overhangs and treetops. Nevertheless, due to its simplicity, several algorithms leverage this technique for ground plane segmentation. Douillard et al. [64] use a mean-based elevation map, i.e., each cell contains the average height of all points, followed by clustering techniques to detect and remove the ground points. The algorithm follows three simple steps: (1) calculates the surface gradients of each cell and classifies protruding objects and ground cells; (2) clusters adjacent ground cells; and (3) corrects artifacts wrongly generated by the gradient computations, e.g., if a cell that was classified as an object has an average height close to its neighboring ground cells, it is changed to a ground cell. Despite presenting good performance results, the proposed solution still needs further improvements to achieve real-time constraints.

The works proposed by Asvadi et al. [65] and Li et al. [66] include, on each cell of the 2.5D elevation grid, the average height and the variance between all points. If both values are lower than a configured threshold, the cell is considered flat and classified as part of the ground plane. The first method was evaluated with the KITTI dataset using point cloud data from a Velodyne HDL-64, and the latter with data from a Velodyne HDL-64E sensor on an Intel i7 dual-core processor running at 3.4 GHz with 8 GB of RAM. Despite this method allowing for very accurate detection of planar objects, it can wrongly classify objects close to the ground as belonging to the ground plane. To address this issue, Meng et al. [67] apply a height difference kernel over each cell and its neighbors, allowing for detecting cells with a slightly higher average height than neighboring cells. Despite improving the detection of low-height objects, extra steps are necessary to address uneven terrains, which significantly increases the overall algorithm’s complexity. There are several applications of these simple techniques, e.g., Tanaka et al. evaluate terrain’s traversability by calculating its roughness and slope using an elevation map created from a HOKUYO UTM-30LX LiDAR mounted on a Pioneer 3DX mobile robot [68].

**Figure 7:**
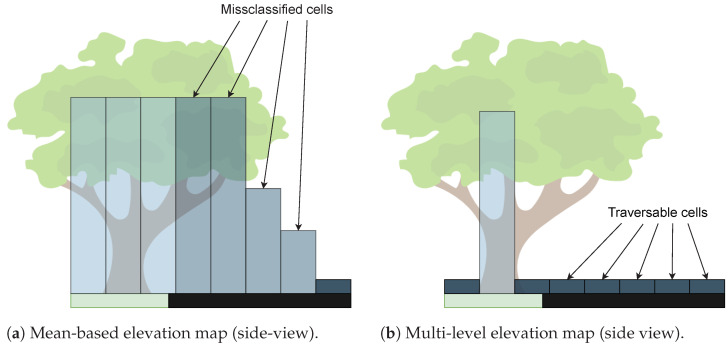
Elevation map techniques with overhangs.

Using elevation maps presents some disadvantages when trying to model free vertical space between points, i.e., in overhanging objects, limiting its utilization in some outdoor scenarios. For instance, using mean-based algorithms in the scenario depicted in Figure 7a causes the system to classify the area below the tree as terrain unable to be driven. To mitigate this, Pfaff et al. [69] proposed an algorithm that allows mobile robots to model vertical and overhanging objects in elevation maps by classifying the surroundings into four distinct classes: (1) regions sensed from above; (2) vertical structures; (3) vertical gaps; and (4) traversable cells. The grid map resulting from this method is depicted in Figure 7b. The algorithm starts by calculating the height variance of each cell, applying a Euclidean clustering technique next. The clustering of points whose distance within the cell is smaller than 10 cm allows for the detection of empty spaces, which is essential for identifying overhangs. Next, when an overhang is detected, only the lowest average height cluster is considered to calculate the average height inside the cell. Despite correctly identifying drivable areas below overhangs, this technique does not allow for the simultaneous modeling of multiple surfaces, i.e., modeling the terrain above an underpass. To solve this issue, the work proposed by Triebel et al. [70] additionally stores the intervals between the detected clusters or surfaces (previously, only the lowest was considered), allowing for the correct representation of all the overhanging surfaces. Both works were evaluated on a Pioneer II AT robot equipped with a SICK LMS291 sensor mounted on an AMTEC wrist PW70.

***Occupancy Grid Maps:*** These algorithms, introduced in the 1980s by Moravec and Elfes, use fine-grained grids to model the occupied and free space in the surrounding environment [71]. The main goal of this technique is to generate a consistent metric map from noisy or incomplete sensor data. This can be achieved by measuring a cell multiple times, so that information can be integrated using Bayes filters [72]. This method is considered highly robust and easy to implement, which is essential for autonomous driving tasks [73]. Stanley, the robot created by the Stanford University that won the Defense Advanced Research Projects Agency (DARPA) challenge in 2005, used occupancy grids for obstacle detection [74]. First, the surroundings are modeled by assuming each cell is in an occupied, free, or unknown state. A cell is considered occupied if the vertical distance between the maximum and minimum height of the detected points exceeds a distance delta. If this verification fails, the area is considered drivable, meaning the ground is successfully detected. In the following editions of the DARPA Challenge, several teams also used occupancy grids for modeling the drivable area, which helped them in finishing the challenge [75,76,77]. Similarly, Himmelsbach et al. [78] proposed the utilization of occupancy grids allied with vehicle position detection mechanisms to accurately segment the ground plane for the object classification tasks, achieving an object classification accuracy of around 96% on the used dataset. By their turn, Luo et al. [79] use a combination of different attributes, such as the average height, the standard deviation of height, and the number of points on each cell, to compute the probability of a cell belonging to the ground plane. This work can achieve a precision ratio of up to 96% with real-time features in light traffic using a Velodyne HDL-32E sensor. The processing unit is composed of an Intel i7-4800 processor running at 2.7 GHz, 16 GB of RAM, and an Nvidia GeForce GTX 1080 graphics card.

### 3.2. Ground Modelling

Ground modeling techniques have been widely adopted and evaluated over the last few years. Typically, these methods consist of expressing the ground’s geometry using a single plane or a grid of planar surfaces and can use one of the three following approaches: (1) Plane Fitting; (2) Line Extraction; (3) or GPR.

**Figure 8:**
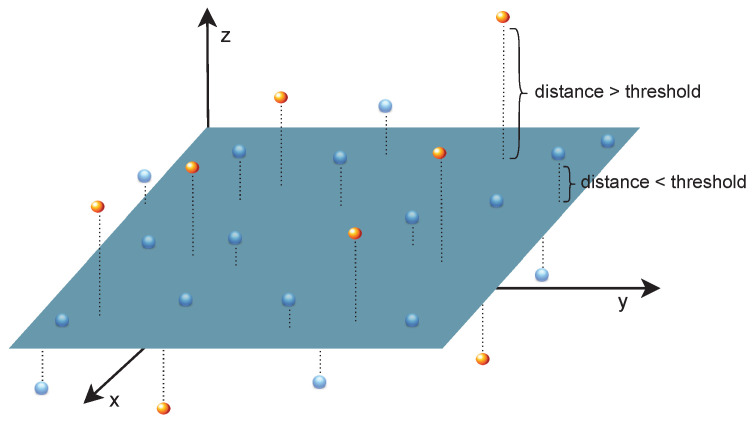
Visual representation of an orthogonal distance classification.

***Plane Fitting:*** The work from Hu et al. [80] identifies the ground plane by fitting the lowest elevation points of each frame and uses the RANdom SAmple Consensus (RANSAC) algorithm to estimate some of the unknown parameters of the plane efficiently. The ground segmentation is then performed based on the orthogonal distance between points and the ground plane (Figure 8). The maximum point-to-plane threshold is then calculated, indicating the minimum distance between a point and a plane for a point to be classified as non-ground. According to the authors, this technique may fail in some situations, reducing the number of ground points and affecting the ground plane estimation’s accuracy. To avoid this limitation, when the number of ground points is severely reduced, a short-term memory technique is used to create a forecast for the current road points based on the previous frame, which is effective on flat surfaces, allowing the algorithm to achieve an effectiveness of around 81% and a precision of 72%. The evaluation was conducted using the KITTI dataset [81], and the experimental setup includes a Linux machine with a Pentium 3200 processor with 3 GB of RAM. Nonetheless, the ground plane is not always flat, thus, the assumption of a single ground plane may result in incorrect classifications. Moreover, noisy or complex data can reduce the RANSAC’s performance.

Similar methods divide point clouds into concentric circles with varying radius centered on the origin [65,82]. This is followed by a gating method to eliminate higher points that are unlikely to represent the ground, and next, a RANSAC-based plane fitting algorithm is applied to the remaining points of each slice to extract ground data. Lim et al. [83] propose a method that uses a Concentric Zone Model-based representation of the point cloud (instead of a Polar Grid Map) to address issues associated with small-sized bins and sparse point distribution. Then, it applies a Region-wise Ground Plane Fitting method based on Principal Component Analysis (PCA), which yields speeds at least twice as fast as the RANSAC algorithm. In the final step of this method, a Ground Likelihood Estimation step is performed, which helps in reducing the number of false positive classifications. Using the KITTI dataset [81], and an Ouster OS0-128 LiDAR sensor, this approach can achieve an $F_{1}$ score of 0.93 with a precision of 92%. The evaluation setup includes an Intel i7-7700K processor, which can execute the segmentation algorithm at a rate of 43.97 Hz. Zhang et al. [84] propose a method for road segmenting based on a planar model. All points with a negative vertical pointing angle are selected, and the RANSAC method is used to estimate the parameters of the plane. Nevertheless, the road is not always perceived as a plane, which results in a precision of 84% and an $F_{1}$ score of 83% with data from a Velodyne HDL-32E LiDAR sensor. This method was tested using an Intel i7-620M processor with a base frequency of 2.66 GHz and achieved an average execution time of 12 ms.

The method of Sun et al. [85] takes into account the presence of barriers on the road and employs two spatial properties of the road boundary, which are adaptable to different heights and road boundary structures. The system uses predictive combination detection to search for road boundary points, and then uses the cubic spline model to fit the road boundary. It is more adaptable to road boundaries with diverse shapes, particularly those with significant curvature variations. Using data provided by an HDL-32E LiDAR sensor, this method can achieve a precision rate of 93% and an accuracy rate of 92%. It was executed on an Intel i7-4790 processor running at 3.4 GHz and 16 GB of RAM, achieving around 36.5 ms to process one point cloud frame. Anandet al. [86] also propose a Plane Fitting method with real-time ground removal, obstacle segmentation, and georeferencing capabilities. In this framework, the ground is subdivided into small square cells that are evaluated based on a series of thresholds. These thresholds have been fine-tuned to prevent the omission of little objects in these cells. Despite its computational simplicity, the suggested ground removal method provides good results for different ground structures. This method was evaluated using the KITTI dataset [81] and could obtain a precision of 92%, a recall of 99%, and an $F_{1}$ score of 0.995. Other datasets were gathered from a Velodyne VLP-32C and two Ouster OS1-64 and OS1-16 LiDAR sensors. Regarding the processing time, with the KITTI dataset, this method can process a frame within 0.0064 s on an Intel Xeon W-2133 CPU with 12 cores running at 3.6 GHz, and 0.074 s using an Intel i5-7200 CPU with four cores running at 2.5 GHz. Using an NVIDIA Jetson TX2 board, which includes a Quad-Core Arm Cortex-A57 processor, the frame processing time is around 0.105 s.

**Figure 9:**
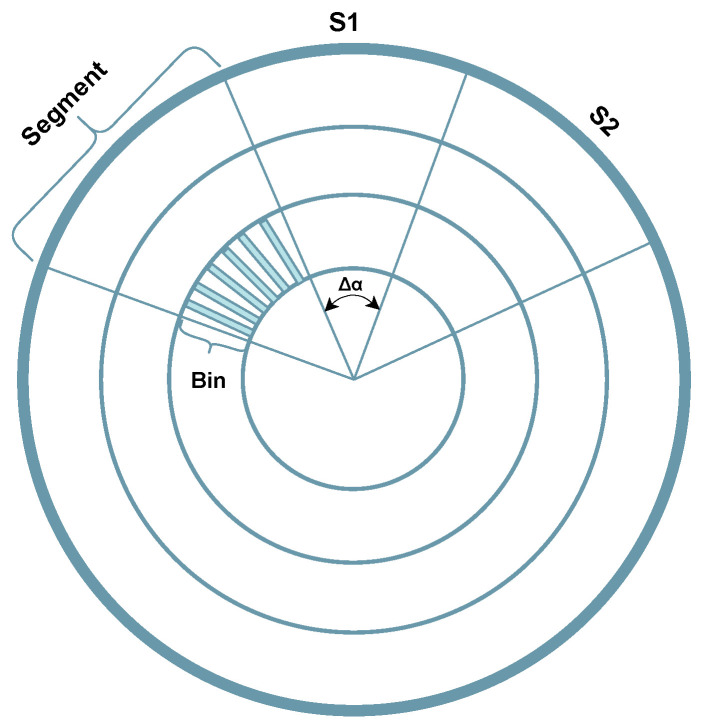
Visual representation of a Polar Grid Map.

***Line Extraction:*** To overcome some limitations of Plane Fitting methods, e.g., the processing time, other works based on Line Extraction have emerged [87,88]. These approaches divide the area under evaluation into several segments of a polar grid map, as depicted in Figure 9, to search for the ground plane. The work from Himmelsbach et al. [87] models the ground using local line fits, and to be considered part of the ground plane, such line fits must satisfy a set of requirements. Considering the slop-intercept equation of a straight line, ***y*** = ***mx*** + ***b***, the slope (*m*) cannot exceed a threshold to exclude vertical structures, and in the case of lower slopes, the line’s intersection with the y-axis (*b*) must not also exceed a threshold to exclude plateaus from the ground plane. After finding the lines that model the ground plane, the distance between the points and these lines is analyzed to determine whether the points are ground or non-ground. This estimation of several local lines/planes allows for greater flexibility in the overall plane fitting. However, when using small regions, this method can be affected by the lack of local points. This strategy can divide the segmentation problem into several smaller and simple fitting problems, thus, using data from a Velodyne HDL-64 sensor, and in a computer with an Intel Core 2 quad-core processor running at 2.4 GHz, it can sometimes achieve real-time performance (0.105 s to process one point cloud frame). However, this approach does not perform well in uneven rugged terrain.

***Gaussian Process Regression (GPR)-Based:*** These methods can be used to estimate and interpolate (to fill gaps in unknown areas) elevation information across the field. They perform well in handling small datasets and have the ability to provide uncertainty measurements on the predictions, demonstrating their applicability in a wide range of autonomous driving features. These approaches require tuning several parameters, which cannot always be obtained beforehand. Therefore, Vasudeva et al. [89] propose a method based on hyper-parameters, which consist primarily of a regression task that, for each segment, solves a supervised learning problem to acquire such parameters. By dividing a complex two-dimensional ground segmentation problem into a large number of one-dimensional regressions, the method can achieve good performance ratios while meeting real-time requirements. However, this approach does not perform well in slowly rising obstacles (e.g., stair steps), as the separation of the ground into independent angular sectors do not keep the general continuity of the ground elevation.

GPR methods assume that the vast majority of data is related to the terrain surface and not to objects, i.e., they assume that there are only a few outliers in the point cloud. Douillard et al. [90] implement a GPR-Based algorithm that employs the Gaussian Process Incremental Sample Consensus (GP-INSAC), which uses a terrain mesh created from the structure of a range image, applied to 3D datasets with several non-ground objects. Following a model-based outlier detection approach, an inlier is a point that belongs to the ground surface, while an outlier is a point that belongs to cluttered non-surface objects that will be removed from the point cloud. This process preserves the features of GPR-based terrain modeling while offering outlier rejection capabilities, albeit being more complex than the previously described methods. This method was evaluated using a 2 GHz dual-core PC using data provided by a Velodyne LiDAR sensor, and was capable of processing a point cloud frame within 0.25 s with a point score of over 80%. To further improve GPR-Based methods, Chen et al. propose a real-time approach to ground segmentation that uses a one-dimensional GPR with a non-stationary covariance function over a circular polar grid map to allow local adaptation to the fluctuations of the ground in each map segment [91]. The method was evaluated on a dual-core Intel P8600 processor running at 2.4 GHz with 2GB of RAM, and using data from a Velodyne HDL-64E S2 LiDAR sensor. It can achieve an accuracy of 0.97 and a processing time of 74.93 ms per point cloud frame.

**Figure 10:**
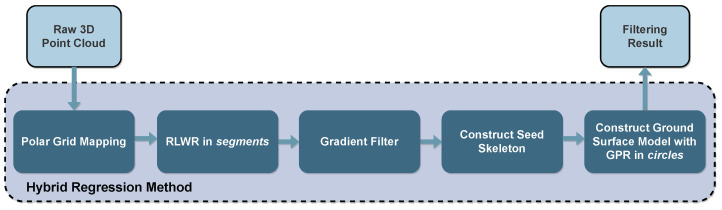
Hybrid Regression Method.

Other works combine GPR methods with different techniques to create more robust solutions. For instance, Lang et al. [92] proposed a GPR method with a non-stationary covariance function for terrain modeling that can locally adapt to the terrain data structure. Authors claim that the adaption method can achieve accurate predictions on both simulated and actual data, decreasing the prediction error from 70% to 30%, and achieving an MSE of 0.059. Time-wise, this method was evaluated using a desktop computer with a 2.8 GHz processor and 2 GB of RAM and took around 44 s to process one frame. The work from Liu et al. [93], whose steps are illustrated in Figure 10, combines the GPR method with a Robust Locally Weighted Regression (RLWR) approach to create a hybrid regression model in which the RLWR is followed by a gradient filter for removing outliers and construct the seed skeleton of the ground plane. The GPR is then used to generate the complete ground surface model based on these initial values. Despite this method achieving an accurate segmentation of the ground surface (nearly 92%), it does not meet the real-time requirements of an automotive application, since it requires around 300 ms per point cloud frame (with the KITTI dataset [81]). The tests were conducted with an Intel Core i7 processor running at 4 GHz on a machine with 16 GB of RAM.

### 3.3. Adjacent Points and Local Features

These techniques explore relationships between neighbor points and local geometric features in a point cloud to classify the surrounding terrain. These relationships include patterns created by LiDAR sensors when generating point clouds, point height differences, and terrain gradients. Algorithms from this group can be divided into Channel-Based, Region-Growing, Clustering, and Range Images.

**Figure 11:**
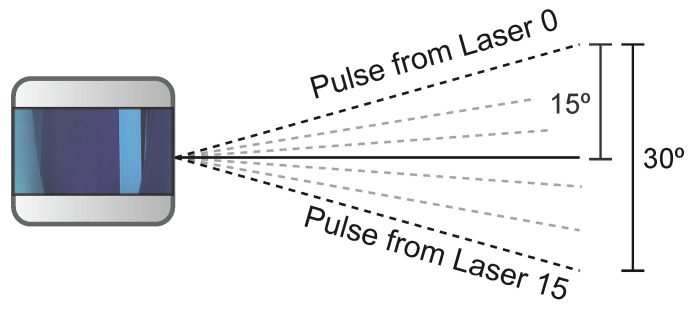
Velodyne VLP-16 side view.

***Channel-Based:*** Channel- or scan-based approaches explore relationships between points in the point cloud that result from patterns in the LiDAR sensor [94]. Taking advantage of the vertically aligned channels in multi-layer LiDAR sensors (Figure 11), Chu et al. [95] separate each frame into vertical lines to analyze points. This method uses a set of geometric conditions, such as height and height gradients, to detect start ground points and threshold points. The start ground points define where the ground begins, and the threshold points represent where an obstacle starts.

**Figure 12:**
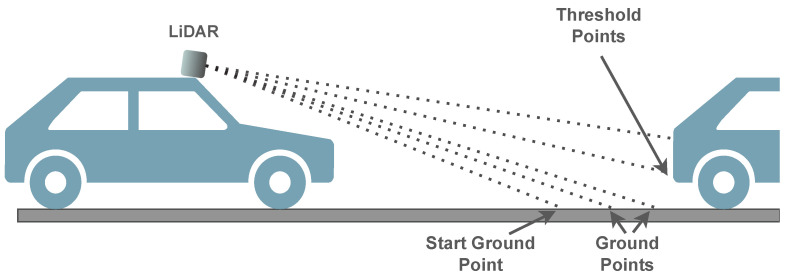
Representation of the channel-based method.

As depicted by Figure 12, points between a start ground point and threshold points are considered ground points since they are all vertically related. Despite performing well, even in bumpy terrains, this method ignores the angular relationship between points and heavily depends on parameterization, both affecting accuracy in complex environments. Other works enhanced the performance and accuracy of this algorithm by adding additional check features to the vertical analysis [96], and by performing a horizontal geometric feature analysis to the previously proposed vertical analysis [97]. Rieken et al. [98] use a channel-based approach for the initial ground classification, followed by the generation of an elevation map with the classified ground points for the filtering step. Finally, it performs a final classification process with the filtered data. The experimental evaluation was performed on a desktop computer equipped with an Intel Core i5-4690 processor running at 3.5 GHz and with 8 GB of RAM, and the point cloud datasets were obtained from a Velodyne HDL-32 sensor. The average processing time of the algorithm is 4.7 ms per frame (213 Hz), indicating that the segmentation process achieves real-time features and can be executed 21 times faster than the sensor output frequency (10 Hz). Despite reducing the dependency on pre-defined parameters, the accuracy of channel-based approaches is still highly dependent on the density of the point cloud. To solve this, Jiménez et al. [94] propose the utilization of an MRF method to enhance the initial channel-based stage. Other works use the concept of sensor rings, which is related to the pattern created by the points generated by a single vertical channel while the sensor rotates, instead of the vertical channel’s alignment [99,100].

**Figure 13:**
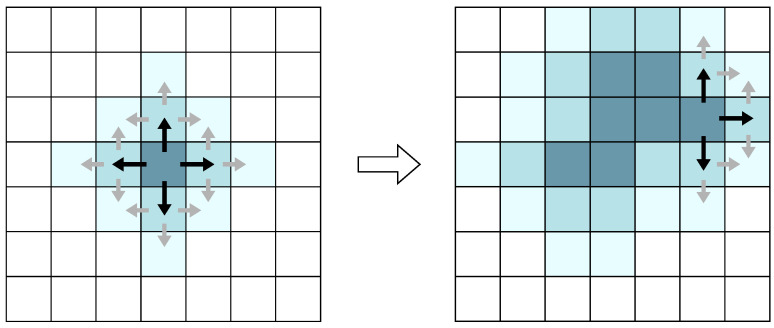
Region-growing labeling based on four neighbours window.

***Region-Growing:*** Region-growing methods perform segmentation by expanding a particular starting seed into a region using pre-defined criteria, usually related to a principle of similarity between those points, as depicted in Figure 13. Moosmann et al. [101] use a graph-based approach where a region-growing algorithm randomly selects the initial seed node, followed by the merging of the neighboring nodes into the same region based on Local Convexity. This algorithm was evaluated with non-optimized code on a Pentium M processor running at 2.1 GHz, and used several scans from an HDL-64 sensor mounted on an experimental vehicle in inner city traffic scenes, achieving good results in urban environments with slightly curved road surfaces. However, the achieved average segmentation time was nearly 250 ms with the selected hardware setup, thus, it cannot achieve the desired real-time performance. Na et al. [102] use a similar region-growing approach, followed by a region-merging algorithm to correct overly partitioned ground regions. Additionally, Kim et al. [103] propose using a weighted-graph structure followed by a voxelization method to improve the region-growing results. Techniques based on Region-growing are simple to implement but suffer from being highly reliant on the selection of a good starting seed and criteria, which may penalize the performance in complex urban environments [104].

***Clustering:*** Similarly to region-growing methods, clustering techniques divide point cloud data into smaller groups. However, unlike region-growing algorithms, some techniques do not rely entirely on analyzing neighboring points. Douillard et al. [90] start by creating a voxel grid, followed by clustering the created voxels based on height averages and variances. In this method, the largest cluster is considered ground and removed from the point cloud. Yang et al. [105] propose a different approach that uses the shape features of adjacent points to assemble points. Since road surfaces are mainly planar objects with low elevations, the ground segments are easily identified. Nitsch et al. [106] compute the point’s surface normal vectors and a quality value based on the weighted average between eight adjacent neighborhood points. Every calculated vector is then compared with the vector perpendicular to the surface below the vehicle. If these vectors are relatively similar, meaning their surface properties are identical to those in the ground below the vehicle, and the calculated quality value is high, the corresponding points are considered good ground candidates. Finally, a euclidean clustering method groups the ground candidates together, where the large clusters are classified as the ground surface. This approach can provide a relatively complete feature analysis, being able to detect 97% of ground points in a simulated data urban scenario and 93% in a simulated rural scenario, using the automatically labeled Virtual KITTI [107]. However, it may not perform as desired in regions with a low point density [94]. Clustering algorithms are considered robust methods that do not require a good selection of seed points/regions, contrary to region-growing-based techniques. However, they can be computationally expensive, especially for large datasets that contain multi-dimensional features [104].

**Figure 14:**
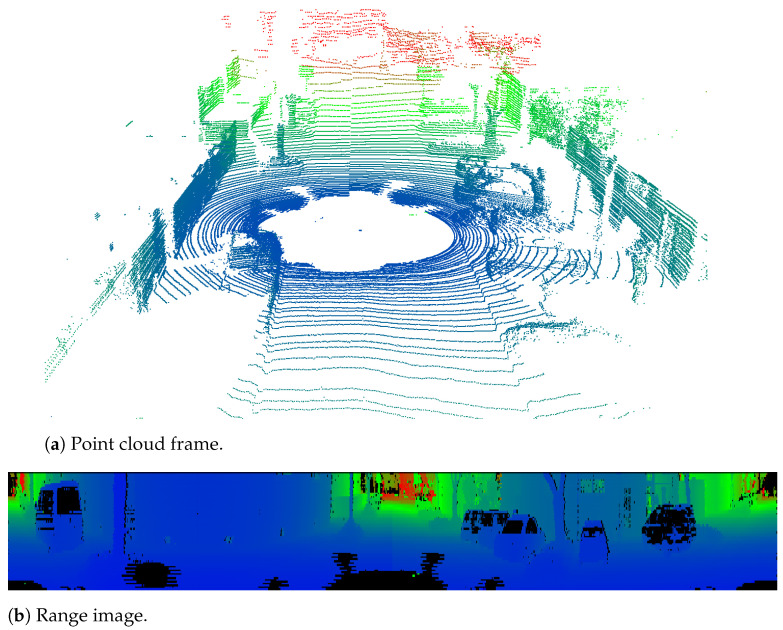
Conversion of a point cloud frame to a range image representation.

***Range Images:*** Typically, LiDAR sensors provide point cloud data in the spherical coordinate system, which is later converted to the cartesian system by the corresponding manufacturer drivers. However, using the spherical coordinate system allows the detection of trigonometric relationships between adjacent points in a point cloud without the associated computational costs of performing these calculations in a cartesian coordinate system [108]. Some approaches make use of the raw data provided by the sensors and transform it directly into a range image, which is a common approach for projecting 3D LiDAR point cloud data into a 2D image [109,110,111,112], as demonstrated in Figure 14. This conversion enables segmentation algorithms to directly exploit the clearly defined neighborhood relations in the 2D image, allowing for real-time segmentation based on angle relations formed by the LiDAR center and the points on the depth image. Bogoslavskyi et al. [109], running on a single CPU core, were able to perform the ground filtering in less than 4 ms using the KITTI dataset. Nonetheless, the method for creating range images is very dependent on the sensor and may affect the accuracy due to the needed sampling process [113].

### 3.4. Higher Order Inference

One of the biggest challenges associated with analyzing LiDAR data is that as the distance to the sensor increases, the points within the point cloud become sparser at longer ranges. This sparsity can lead to false classifications in point cloud segmentation methods, representing a significant drawback in automotive applications. Higher order inference methods have proven useful in multiple computer vision tasks, e.g., semantic segmentation [114], which has led to their recent implementation in LiDAR systems to overcome problems related to ground segmentation of sparse point cloud scenarios. This group of methods includes methods based on the MRF and CRF algorithms.

***Markov Random Field (MRF):*** An MRF model can be seen as an undirected graph, where nodes represent random variables and edges represent desired local influences between pairs of nodes. In the case of LiDAR data, the points are the nodes of the graph and the edges can be modeled using the height values. Guo et al. [115] and Byun et al. [116] suggest the combination of an MRF with a Belief Propagation (BP) algorithm to identify the drivable area, even at high distances. Nonetheless, these approaches still face detection problems when driving in rough terrains. The work from Zhang et al. [117] tries to improve these approaches to be used both in rough and uneven terrains. It implements cost functions to generate probabilistic ground height measurements, creating models that compensate the loss of information due to partial obstruction from closer objects. Based on this information, combined with a BP algorithm, a multi-label Markov network is used for the ground segmentation task. The proposed method shows promising results in sparse point cloud distributions, achieving false positive rates as low as 2.12% in complex off-road environments. However, the average processing time in the mentioned scenario was above 1 s using an Intel Core processor running at 3.2 GHz, which makes its utilization hard in embedded perception systems with real-time requirements. Other works use similar approaches combined with height histograms to estimate the ground height range, followed by the MRF model to refine the labeling of ground points [118].

**Figure 15:**
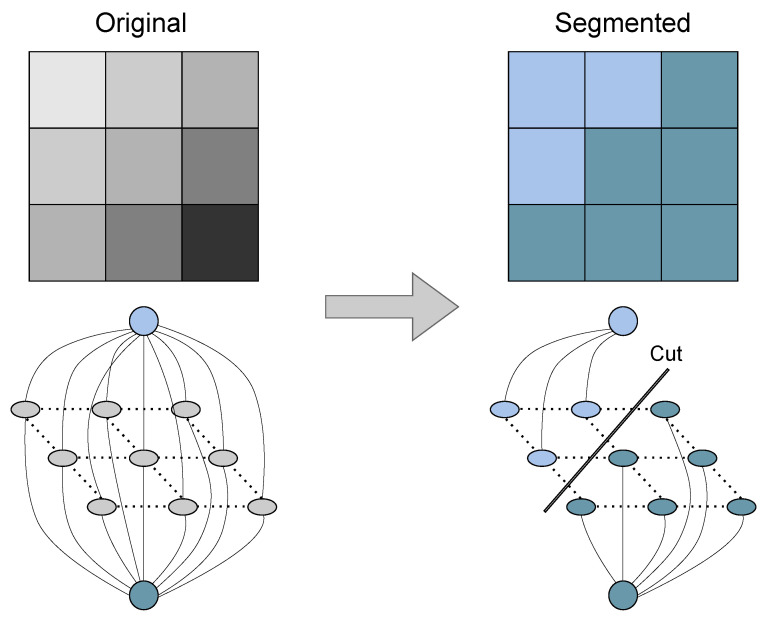
Graph Cut segmentation method.

Huang et al. [119] propose an algorithm that aims at solving the high computational requirements of MRF-based methods. This algorithm starts by performing a coarse segmentation based on a ring-based elevation map where data points are arranged in rings whose diameters are proportional to the distance to the LiDAR sensor. Since this type of algorithm assumes that the ground in the grid is flat, which is not the case in most real-world scenarios, an optimization algorithm based on spatiotemporal adjacent points is applied. Thereafter, to perform a more refined segmentation, the point cloud is converted into a range image where each point is projected as a graph node to create an MRF model, unlike the previous approaches that convert grids into graph nodes [94,114]. Nevertheless, MRF methods based on iterative implementations, such as the BP algorithm, are usually computationally expensive and time-consuming to perform the segmentation task. To mitigate this, this approach initializes the network with information regarding the high-confidence obstacle points and ground points captured from the segmentation algorithm, which helps in reducing the computational complexity and avoids convergence problems that usually exist in other implementations. Finally, the algorithm applies the graph cut segmentation technique, depicted in Figure 15, to solve the model, which helps in achieving the desired fine segmentation results. Due to the reduction in the algorithm’s complexity, the proposed work can achieve an average processing time of 39.77 ms with a single core from an Intel i7-3770 processor while using the KITTI dataset containing data from a Velodyne HDL-64E.

***Conditional Random Field (CRF):*** CRF is a subset of MRF that labels sequences of nodes given a specific chain of observations, which improves the ability of capturing long-range dependencies between them. Rummelhard et al. [120] propose the addition of spatial and temporal dependencies to CRF to model the ground surface. Their method divides the environment into different interconnected elevation cells, which is influenced by local observations and spatio-temporal relationships. The temporal constraints are incorporated into the segmentation data using a dynamic Bayesian framework, allowing for a more accurate modeling of ground points. The proposed method was first tested on an Intel Xeon W3520 processor running at 2.6 GHz with 8 GB of RAM and with a Quadro 2000 graphics card with 2 GB of video memory, achieving a frame processing frequency of 6.8 Hz with data from a Velodyne HDL-64E sensor. However, the authors claim that with experimental platforms such as Tegra X1 and K1, the algorithm achieved real-time performance. Similarly, and following the approach of using CRF methods in the segmentation of data from digital cameras [121], Wang et al. [122] model ground data by representing the CRF in a 2D lattice plane. Next, it uses the RANSAC on the created plane to extract the ground points. Despite presenting good segmentation results, it requires several iterations to correctly extract the points in uneven terrains, which heavily compromises real-time performance. Additionally, these methods are very computationally-hungry, which makes them unsuitable for real-time scenarios unless specialized hardware acceleration is used.

### 3.5. Learn-Based

Learn-based methods have been widely applied to camera vision systems, achieving good results in a variety of segmentation tasks, including ground segmentation. Nonetheless, due to the inherent benefits of deploying LiDAR sensors in the perception system of the car, several learn-based methods applied to point cloud data began to emerge [123]. Back in 1988, Pomerleau [124] introduced Autonomous Land Vehicle In a Neural Network (ALVINN), a 3-layer back-propagation network designed for the task of road following that was trained from multiple simulated road images. Despite the solution not allowing the vehicle to follow the road at high speeds, primarily due to computational limitations at the time, it was good enough to validate this new approach and to demonstrate that learning-based methods could become potential solutions for autonomous driving tasks. The success of ALVINN has triggered the utilization of machine learning algorithms applied to LiDAR data, which, based on the success of these approaches, several algorithms have emerged and many more are currently being released. Thus, it makes it very hard to compile all the existing information in this section. Nonetheless, we provide the most significant algorithms that made great contributions to this topic.

PointNet [125] is a unified architecture for different applications, e.g., object classification and segmentation, scene semantic parsing, etc. Despite showing promising results resistant to input perturbation and corruption, by design, this architecture is only suitable for small-scale point clouds. This is mainly due to the weak learning capabilities of local features, affecting its performance in large-scale complex point clouds and limiting its capacity to identify fine-grained patterns. Aiming at improving the previous work, PointNet++ [126] implements a hierarchical framework to discover the multi-scale spatial contextual elements, which improves performance in large-scale point clouds. Nonetheless, the hierarchical structure increases the overall computing complexity. Other emerging approaches, such as the Pointwise CNN [127], focus on pointwise convolution operations applied to each point in a point cloud, which achieves good accuracy results in the semantic segmentation and object recognition tasks. SPGraph [128] organizes the point cloud using a data structure known as the Super Point Graph (SPG), which is created from partitioning the scanned image into geometrically homogeneous elements. The evaluation was conducted on a 4 GHz CPU and GTX 1080 Ti GPU over two different datasets, Semantic3D and the d Stanford Large-Scale 3D Indoor Spaces (S3DIS). PyramidPoint [129] employs a dense pyramid structure that provides a second glance at the point cloud, allowing the network to revisit distinct layers formed from other network levels, which enables feature propagation. However, these methods require expensive nearest-neighbor search algorithms in sparse space and are inefficient when dealing with outdoor and automotive point clouds. Nonetheless, based on their success, these principles have been used to help solve the ground segmentation problem. The experiments were performed with three different types of datasets, i.e., aerial, terrestrial, and mobile laser scanners, on a single NVIDIA TITAN RT GPU.

**Figure 16:**
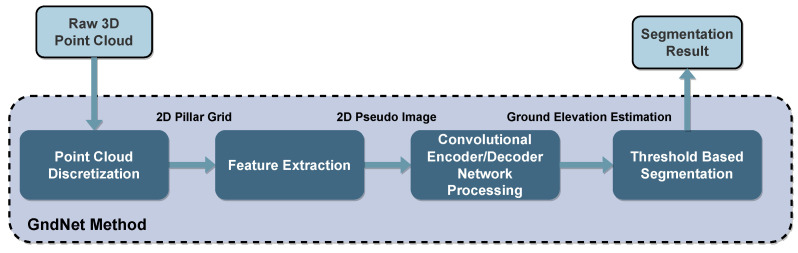
Overview of the GndNet algorithm.

Paigwar et al. [130] developed the GndNet, whose steps are shown in Figure 16. The 3D point cloud ground plane elevation is estimated in a grid-based representation, and the points are divided into ground and non-ground categories. The method starts by transforming the point cloud into an evenly spaced 2D grid, producing a series of point-populated pillars. Then, to extract the features contained in each non-empty pillar, a simplified version of the PointNet method is used [125,126], which generates a pseudo-image of the grid. Then, a 2D convolutional encoder-decoder network processes this image to produce a high-level representation. However, for training the network, it is necessary to use a labeled ground elevation map dataset. To solve this, the authors used the SemanticKITTI [81,131] and modeled the ground plane with a CRF method to generate a ground-truth elevation map suitable for the required training. This allowed GndNet to achieve a mean intersection over union (mIoU) value of 83.6%, while providing real-time performance with an average processing time of 17.98 ms per frame.

Despite the maturity of point-wise approaches, new approaches start by representing point clouds as structures before applying the learning-based algorithms, which helps in achieving more efficiency while requiring less computational resources [132]. For instance, VoxelNet [133], discretizes point clouds into voxels before using a 3D CNN. The proposed Voxel Feature Encoding (VFE) layer integrates feature extraction and bounding box prediction into a single stage, allowing the algorithm to achieve an inference time of around 33 ms and a precision of up to 89.6% on a processing system composed of a TitanX GPU with a CPU running at 1.7 GHz. On the other hand, SPLATNet [134] interpolates point clouds into permutohedral sparse lattices before executing a 3D CNN, enabling a straightforward mapping of 2D information into 3D and vice-versa. Cylinder3D [135] builds a cylindrical partitioning and then uses an asymmetrical 3D convolution network that enables it to reach an mIoU of 61.8% using the SemanticKITTI dataset, which provides data from a Velodyne HDL-64E LiDAR sensor. PointPillar [136] is a novel encoder method that uses PointNet [125,126] to create pillar representations from the input point clouds. This representation can then be used by a 2D convolutional architecture to process it into higher-level representations for the identification steps. Additionally, this method can achieve real-time performance, with inference taking 16.2 ms per frame on a computing system composed of an Intel i7 processor and a 1080ti GPU.

SqueezeSeg [137,138,139] is another work that uses these particular structures, where a 2D spherical projection point-wise label map is used and subsequently refined by a CRF implemented as a recurrent layer. Its most recent version, SqueezeSegV3, supports two different implementations that can achieve different performance results. SSGV3-53 reaches an mIoU of 52.2% with 11 frames per second and SSGV3-21 achieves an mIoU of 48.8% with 16 frames per second. RangeNet++ [140] is a deep-learning method that uses range images and 2D convolutions, followed by GPU-accelerated post-processing methods, to recover consistent semantic information for complete LiDAR scans. Similarly to SqueezeSeg, RangeNet presents two implementation versions, where RangeNet21 can achieve an mIoU of 47.4% with 20 frames per second and RangeNet53 an mIoU of 49.9% with 13 frames per second. All tests were conducted on GPU-based setups with the KITTI dataset. Finally, PolarNet [141] uses a polar Bird’s-Eye-View (BEV) representation, which balances the points across grid cells in a polar coordinate system, allowing it to achieve an mIoU of 54.3% with 16.2 frames per second on the SemanticKITTI dataset.

Several works use CNNs to identify and segment obstacles within a LiDAR point cloud. However, performing learn-based algorithms on LiDAR data requires a significant amount of processing power which often translates into very time-consuming tasks. Consequently, Lyu et al. [142] proposed an efficient FPGA-based solution that can process each LiDAR scan within 16.9 milliseconds using the KITTI dataset [81,131] as a test bed. Velas et al. [143] used CNNs to perform ground segmentation, considerably reducing the algorithm’s processing time when compared with previous learn-based approaches, but failing to achieve real-time performance in a CPU implementation (Intel i5-6500 processor). The best performance results were achieved with GPU acceleration (NVIDIA GeForce GTX 770), where different network topologies were tested. With a topology consisting of 5 convolutional layers plus a single deconvolution, the achieved processing time of one Velodyne HDL-64E frame is, on average, less than 7 ms. Nevertheless, this method can only be applied to ground point segmentation rather than other segmentation tasks. Zhang et al. [144] introduced ShellNet, a method for applying deep learning to 3D point clouds. It is built on a convolution operator that represents point sets with locally created spherical shells. This method can result in fast point cloud segmentation and outperform other methods in terms of accuracy. However, the lack of labeled training data still poses a significant obstacle to the algorithm’s performance in many scenarios.

Shen et al. [145] propose a method based on the jump-convolution-process (JCP) to solve the problems faced by segmentation algorithms in handling complicated terrains, and the excessive processing time and memory requirements. The method starts by projecting onto an RGB image the point cloud previously labeled by an improved local feature extraction algorithm. The pixel value is then initialized with the point’s label and continuously updated using image convolution. Finally, it is used the convolution process with a jump operation to perform operations only on the low-confidence points filtered by the credibility propagation mechanism, which reduces the execution time. This method shows good performance results in different environments, achieving an average processing time of 8.61 ms and 15.62 ms when dealing with 64-beam and 128-beam LiDAR data, respectively.

**Figure 17:**
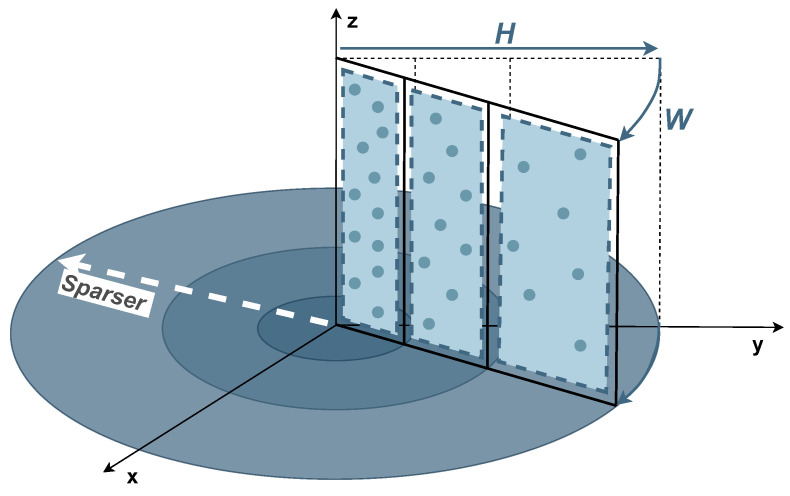
BEV sector partition representation.

He et al. [132] propose SectorGSnet, an end-to-end DNN framework designed to perform ground segmentation of outdoor LiDAR data. The algorithm starts with a sector encoder module in which the 3D point cloud is divided into different sections based on a BEV sector partitioning method, as depicted in Figure 17. The point cloud is represented in a circular region, followed by its division into equal slices (W) and radial line segments (H) that are dynamically adjusted along the radius so that the points become more evenly distributed across the grid.

After partitioning the point cloud, some features, e.g., point coordinates and intensity, are aggregated in each sector using a simplified version of the PointNet [125,126] encoder, resulting in the generation of a sector feature map. Then, the sector encoder module receives the sector feature map to process it using the UNet CNN to predict the sector labels. In the final stage, a sector-to-point restoration is performed to reconstruct the segmented point cloud. All models were trained on a desktop computer with an NVIDIA GTX2080Ti graphics card and an Intel i7 6700k processor, resulting in inference results at 170.6 Hz.

## 4. Discussion

When choosing a ground segmentation algorithm, it is crucial to understand their most significant differences and features that best suit the requirements of the final application. Table 1 summarizes a qualitative comparison of current state-of-the-art ground segmentation methods regarding the following metrics: real-time features, computational requirements (which are sometimes dictated by the algorithm’s complexity or steps to achieve real-time capabilities), the segmentation immunity, and the ability to deal with rising obstacles and regions, uneven ground, and sparse data in the point cloud. This comparison is made solely based on available information retrieved from respective literature, whose experimental setups are of the responsibility of respective authors.

***Real-time:*** Within an autonomous driving scenario, it is mandatory for the perception system to process and understand the surrounding environment in real time, which means that, for a given sensor or a set of sensors, the steps of the LiDAR processing stack must be performed within a known period of time so that the driving decisions can be taken within a safe time frame. A high-resolution LiDAR sensor can generate millions of data points. For instance, the Velodyne VLS-128 can produce up to 9.6 M points per second in the dual-return mode, with frame rates varying from 5 Hz to 10 Hz. In a typical operation, this sensor can be configured to produce, on average, a point cloud of 2,403,840 points per second (240,384 points at 10 Hz), which means that such an amount of data must be processed in under 100 ms across all software stack layers. Regarding the ground segmentation tasks, with a few exceptions, both 2.5D Grid-based and Ground Modelling methods can achieve real-time processing. For the algorithms based on the multi-level approach [69,70], this information could not be retrieved. Due to their complexity, most of the GPR-based methods [90,92,93] and one Plane-fitting method [80] are unable to provide the desired real-time features.

Concerning the algorithms based on adjacent points and local feature extraction, i.e., Region Growing [101], Clustering [90], one from Range Images [111], and one from Channel-based [94], they do not provide real-time (likely due to lack of optimizations or hardware setup used), while the remaining methods [94,95,96,97,98,100,109,110,112], are considered real-time. Regarding the Higher Order Inference [115,116,117,118,119,120,122] and Deep learning [125,126,127,128,129,130,132,133,134,135,136,137,138,139,140,141,142,143,144] methods, they naturally do not provide real-time ground segmentation due to the overall algorithm’s complexity. However, when resorting to hardware acceleration, e.g., based on GPUs [120,130,133,136,140] or FPGA [142], or taking advantage of coarse segmentation results from local feature extraction [29], some solutions can meet real-time requirements.

***Computational Requirements:*** When deployed in automotive applications, the computational requirements associated with ground segmentation methods are a crucial metric to consider, mainly because the perception system is often composed of embedded processing environments that try to minimize the available hardware resources. Methods based on Elevation Maps present the lowest computational requirements as they analyze a 2D tessellated representation of the surroundings, instead of the entire 3D representation. Regarding the other 2.5D grid-based approaches, Multi-level algorithms require extra classification steps, while Occupancy Grids require the interpolation of data and the use of Bayes filters, which inherently increases the memory and computational needs in both cases. Likewise, GPR-based and Plane Fitting methods (based on RANSAC) rely on iterative approaches, consequently increasing the memory requirements. Additionally, GPR methods require complex calculations to process the point cloud, which further increases the computational needs. On the other hand, Line Extraction approaches feature a lower resource consumption than the remaining Ground Modelling methods since they divide the point cloud into a polar grid map, simplifying the required computations. Concerning the Adjacent Points and Local Features methods, Channel-based and Range Image approaches are not considered very computationally intensive. However, they are based on the analysis of geometric conditions between points, which can represent the need for specialized hardware, such as a floating-point unit for trigonometric calculations. On the other hand, despite their simplicity, Clustering and Region-Growing methods require very iterative operations, which can represent high memory requirements especially for large point clouds with points containing multiple features, e.g., target’s reflectivity. On the other side, Higher Order Inference methods feature high computational requirements due to the associated complex mathematical computations and respective iterative steps, e.g., the BP algorithm, which translates into high memory requirements. Finally, among all methods, Learn-based approaches demand the highest computational needs. This is mainly caused by the extensive complex computations, significant memory utilization, and sometimes specialized hardware, e.g., GPUs, generally associated with CNN implementations.

***Segmentation Immunity:*** Segmentation immunity refers to the algorithm’s susceptibility to under- and/or over-segmentation, which corresponds to either too coarse or too fine segmentation, respectively. When the under-segmentation occurs, the points belonging to different objects are merged into the same ground segment. On the other hand, with over-segmentation, a single object can be represented by several clusters. In many applications, under-segmentation is considered a more severe issue than over-segmentation, since the wrong classification of the ground plane can lead to safety issues to the autonomous vehicle. 2.5D Grid-based Elevation Map algorithms tend to suffer both from under- or over-segmentation, which can highly affect the overall algorithm’s performance and accuracy. That happens especially when the ground is significantly sloped or curbed. Ground Modelling’s Line Extraction and Plane Fitting [82] methods tend to slightly suffer from under- or over-segmentation. However, GPR-based [91] and Plane Fitting [83] are able to overcome under-segmentation issues. Adjacent Points and Local Features algorithms typically are susceptible to under- or over-segmentation, except for [103], which is immune to over-segmentation, while [97,105] are immune to both under- and over-segmentation.

***Performance with Rising Obstacles and Regions, Uneven Ground, and Sparse Data:*** In a real-world driving scenario, the ground is not flat and, rising obstacles, regions, and uneven ground usually represent a significant challenge to ground segmentation algorithms. Additionally, dealing with sparse point clouds can lead to performance loss or algorithm inability to solve the segmentation task. Therefore, to assess the versatility and safety of a method in different situations, it is crucial to evaluate the segmentation performance with rising obstacles, slopped or rough terrains, and sparse data. Regarding 2.5 Grid-based methods, since the ground is modeled into a grid where each cell represents a small region of the ground plane, almost all of them can perform well with rising obstacles and uneven ground surfaces [67,68,69,70]. However, they can be unpredictable when dealing with sparse data. The Ground Modeling approaches based on GPR algorithms can suffer from insensitivity to these slowly rising obstacles [91].Since the ground division in independent angular sectors does not guarantee the general ground elevation continuity, some obstacles, such as stair steps, may be classified as ground. Nonetheless, the other approaches, i.e., Line Extraction [88], and Plane Fitting [83,86], can handle these objects properly. Concerning the uneven ground regions, some Ground Modelling approaches cannot handle them properly [80,87], e.g., the Plane Fitting method [80] uses RANSAC in order to estimate the ground plane. Therefore, the assumption of a single ground plane leads to false classifications and complex data can degrade the RANSAC performance. Nonetheless, most GPR-based algorithms [90,92,93], and one Plane Fitting method [82], can achieve good segmentation results when handling sparse data.

From the Adjacent Points and Local Features methods, most of them can handle rising obstacles and regions, e.g., Channel-based [94,95,96,97,98], Range Images [109,110], Clustering [106], and region Growing [104]. Regarding handling uneven ground, some Channel-based approaches [94,97] can perform well in sloped environments, where [94] developed algorithms specialized in handling sloped terrains and sparse data, and [97] proved to achieve good results with sloped and flat environments. Some methods [90,94,109,110] can also perform well in sparse data, highlighting the works of [94,109,110] that developed methods especially for sparse point clouds. Some Higher Order Inference MRF methods can perform well in rising obstacles and regions [115,116], as there are also others that manage to obtain good results on both sloped and rough terrains [115,117]. Unfortunately, no other information could be found on the other metrics for the CRF algorithms. While handling with sparse point clouds, some MRF methods [115,117] and one CRF method [120] can work adequately with sparse point clouds. Deep Learning methods generally obtain the most accurate results in terms of ground segmentation on well-trained environments, being able to perform well in sloped and rough terrain, as well as in dealing with sparse data. However, the lack of labeled datasets for the training phases, the time-consuming conversions between 3D point cloud data and network input data, the need for powerful hardware support, and the complexity involved in developing algorithms, usually hampers the development of CNNs for the segmentation ground tasks.

***Future trends of ground segmentation methods:*** Ground and object segmentation is an important task in autonomous driving applications, where the algorithm’s performance and the real-time aspects are the most critical requirements when building the perception system of the vehicle. Automotive LiDAR sensors are also becoming mainstream, thus, several algorithms and approaches to process point cloud data are constantly emerging. With the success of learn-based approaches in terms of accuracy and performance, it is expected that future solutions will adopt CNNs to perform the ground segmentation tasks [123]. With the constant development of technology and research around these topics, currently challenges of CNNs with LiDAR data applied to automotive, e.g., the lack of datasets [146], the high time-consuming training phases, the requirement for powerful computing systems, and the algorithm’s complexity, are slowly being mitigated. Despite perception systems being including more sensors and LiDAR devices providing more resolution data at higher frame rates (which inherently increases the amount of data to be processed), it is expected that in the near future learn-based solutions with lower hardware requirements will perform the ground segmentation steps with reduced processing times.

## 5. Conclusions

An autonomous vehicle requires a good perception system to successfully navigate the environment, required for object identification and obstacle avoidance, and the ground plane and road limits detection tasks. Detecting and classifying the drivable area is undoubtedly a crucial task that requires extracting information from the point cloud to precisely detect common road boundaries. However, this is only possible with fast and efficient ground segmentation methods that can deliver real-time features, and choosing the best that suits the final application will surely affect the way the vehicle moves around. This article presents a lightweight survey of existing methods to detect and extract ground points from LiDAR point clouds. It summarizes the extensive literature and proposes a comprehensive taxonomy to help understand the current ground segmentation methods that can be used in automotive LiDAR sensors. The proposed categories are as follows: (1) 2.5D Grid-based methods, (2) Ground Modeling, (3) Adjacent Points and Local Features, (4) Higher Order Inference, and (5) Learn-Based methods, where more solutions are likely to exponentially emerge in the very near future. Moreover, and to understand the main differences between them, this article also includes a qualitative comparison where important metrics, such as real-time, computational requirements, segmentation immunity, algorithm’s performance in different conditions, are discussed.

## Figures and Tables

**Table 1:** Qualitative comparison between existing ground segmentation methods.

	*Metric*	*Real-Time*	*Computational Requirements*	*Segmentation Immunity*	*Performance with Rising Obstacles and Regions*	*Performance with Uneven Ground*	*Performance with Sparse Data*
*Method*							
**2.5D Grid-based**	Mean-Based [64,65,66,67,68]	✓ [66,68]; ✗ [64]	Low	Under-/over-segmentation	Good [67,68]	Good [67,68]	-
	Multi-level [69,70]	-	Medium	-	Good	Good	-
	Occupancy Grid [74,75,76,77,78,79]	✓ [74,75,76,77,78]; ✗ [79]	High	Under-segmentation [74]	Good [79]	-	-
**Ground Modelling**	GPR-based [90,91,92,93]	✓ [91]; ✗ [90,92,93]	High	Under-segmentation [91]	Insensitive to slowly rising obstacles [91]	Good [91,93]	Good [90,92,93]
	Line Extraction [87,88]	✓ [87]	Medium	Slight Over-/under-segmentation	Good [88]	Good [88]; Bad [87]	-
	Plane Fitting [80,82,83,84,85,86]	✓ [82,83,84,85,86]; ✗ [80]	Medium/High	Prone to over-segmentation [82]; Under-segmentation [83]	Good [83,86]	Good [83,86]; Bad [80]	Good [82]
**Adjacent Points and Local Features**	Channel-based [94,95,96,97,98,99,100]	✓ [95,96,97,98,100]; ✗ [94]	Medium	Under/over-segmentation [94]; Under/over-segmentation [97]	Good [94,95,96,97,98]	Good [94,97]	Good [94]
	Range Images [109,110,111,112]	✓ [109,110,112]; ✗ [111]	Medium	Prone to over-/under-segmentation; Over-segmentation [109,110]	Good [109,110]	-	Good [109,110]
	Clustering [90,105,106]	✗ [90]	Medium/High	Under-/over-segmentation [105]	Good [106]	-	Good [90]
	Region Growing [101,102,103,104]	✗ [101]	Medium/High	Small over-segmentation [101,102,104]; Small under-/over-segmentation [103]	Good [104]	-	-
**Higher Order Inference**	MRF [115,116,117,118,119]	✓ [119]; ✗ [117]	High	-	Good [115,116]	Good [115,117]; Bad [116]	Good [115,116]
	CRF [120,122]	✓ [120] (with GPU); ✗ [122]	High	-	-	-	Good [120]
**Deep Learning**	CNN [125,126,127,128,129,130,132,133,134,135,136,137,138,139,140,141,142,143,144]	✓ [130,133,136,140] (with GPU), and FPGA [142]; ✗ [143]	High/Very High	-	Good	Good	Good
